# Expressive Existential Psychoeducational Intervention Treatment for Maladaptive Sexual Behavior in a Boy with Trisomy 21 Syndrome

**DOI:** 10.1007/s10508-025-03186-3

**Published:** 2025-06-04

**Authors:** Stefano Federici, Elia Mosconi, Maria Elena Rossler, Vittorio Mossuto

**Affiliations:** 1 Department of Philosophy, Social & Human Sciences and Education, University of Perugia, Piazza G. Ermini, 1, 06123 Perugia, Italy; 2 LoveLife APS, Association for the Social Promotion of the Sexual Health of People With Disabilities, Perugia, Italy

**Keywords:** Psychoeducational intervention, Intellectual and developmental disability, Maladaptive sexual behavior, Down syndrome

## Abstract

The objective of this research was to provide a case report on the efficacy of an expressive existential psychoeducational intervention (EEPI) to reduce maladaptive sexual behaviors in an adolescent with intellectual disability. The method was based on a prospective longitudinal single-case assessment, using a descriptive approach, to assess the effectiveness of the intervention through the analysis of qualitative data obtained during a structured interview. We report the case of G, a 17-year-old male high school student with Trisomy 21 syndrome and moderate intellectual disability. G’s parents requested an intervention to help their son acquire better adaptive sexual behavior skills—regarding appropriate management of masturbation, distinguishing between a public and a private place, managing aggression, and oppositional behavior—to avoid the risk of school exclusion and to improve his quality of sexual, emotional, and social life. Data on adaptive and maladaptive behaviors were collected using the Vineland-II and on sexual knowledge and behavior using the Sexual Knowledge and Behavior Assessment Tool. Both interviews were administered at the beginning (t1) and end (t2) of treatment. The Vineland-II was also administered 3 months after treatment (t3). The main outcomes of this case study concerned: (1) public/private management; (2) anger and aggression management; and (3) management of an autonomous masturbation path. Comparing the results of the pre- (t0) and post-treatment (t2) interviews and the Vineland-II administration at follow-up (t2), a significant reduction in G’s maladaptive sexual behavior was observed. The EEPI, when carried out from a preventive perspective, was effective in the case of an adolescent with intellectual disability. The advantages and disadvantages of the EEPI model are discussed by comparing it with other pharmacological and behavioral treatments.

## Introduction

According to the International Classification of Functioning, Disability and Health (ICF), “disability is characterized as the outcome or result of a complex relationship between an individual’s health condition and personal factors, and of the external factors that represent the circumstances in which the individual lives” (World Health Organization [WHO], 2001, p. 17). In other words, disability is determined not only by biological or social factors of human functioning independently, but by a triadic reciprocal causation among the health conditions, the environment, and the personal factors which affect an individual’s life.

This model of disability replaced the etiological linear perspective that, from an altered state of health, leads to disability, as the medical model of the WHO’s (1980) first disability classification claimed. This context-dependent view of human functioning and disability implies that any health intervention to prevent (e.g., providing in school a masturbation policy and training on the issue to prevent inappropriate masturbation in the classroom or in school toilets), reduce (e.g., managing of aggression and oppositional behavior may avoid exclusion from school), or overcome (e.g., by increasing the quality of sexual and emotional skills of a person with intellectual disability, which may overcome social exclusion from peers) must include interventions to both neutralize social and physical barriers and improve psychological well-being as an integral part of the care process. This complexity that characterizes any intervention in favor of human health and personal well-being increases then enormously when it is aimed at the sexual health of people with disabilities. In fact, promotion of the sexual health of people with disability finds obstacles not only and not so much because of their individual functioning conditions, but because of the barriers that the context (family, social, and cultural) poses to the healthy development of their sexuality as people with disability (Federici, 2002; Federici & Lepri, 2021; Shakespeare et al., 1996).

A recent paper by Pérez-Curiel et al. (2023) reviewed the evidence on how the rights of people with intellectual disability in relation to sexuality and reproductive health—as stated by the articles 23 (right to home and family) and 25 (health, specifically sexual and reproductive health) of the United Nations (UN) Convention on the Rights of Persons with Disabilities (CRPD; UN, 2006)—have been received around the world. From the 151 articles included for review, it is clear that there are still many barriers that prevent people with intellectual disability from fully exercising their right to sexuality, reproductive health, and parenthood, barriers preventing them from enjoying a good quality of life. The barriers limit the functioning of people with intellectual disability, thus restricting the individual’s performance (WHO, 2001).

Precisely because of the difficulty on the part of UN members in transposing and guaranteeing the rights to sexual health to all people, the WHO has repeatedly issued documents, planned strategies, mobilized political will, and worked to ensure that all people can achieve the highest standards of sexual and reproductive health and rights (WHO, 2006; 2009, 2010, 2015; WHO & BZgA, 2010). In August 2023, the WHO and The European Parliamentary Forum for Sexual and Reproductive Rights signed a Memorandum of Understanding (WHO, 2023) to promote health and rights through core functions of legislation, advocacy, accountability, and budget allocation within the European Union. Alongside the large governmental institutions, several non-governmental organizations worldwide (e.g., the International Planned Parenthood Federation, https://www.ippf.org/about-us) and in Italy (e.g., LoveLife APS, https://www.love-life.it; Lovegiver, https://www.lovegiver.it) provide services and advocate for sexual and reproductive health and rights for all, promoting the principles of the CRPD and WHO.

However, ensuring the sexual and reproductive health of people with intellectual disability also involves protecting them such that the exercise of their right to sexuality is expressed in socially acceptable ways, helping them avoid problematic, maladaptive, or inappropriate behaviors. This means that sexual and affective expression takes place in ways that are expected of a person in a given context, in relation to their age, gender, and cultural group. In fact, when problematic/maladaptive behaviors involve the sphere of sexuality draws hate and social condemnation (Lockhart et al., 2009). This implies that the person has acquired, learned, enacted, and practiced in daily life the set of conceptual, social, and practical skills proper to their cultural context and consistent with their developmental age; in a few words, that the individual manifests appropriate adaptive behavior (American Psychiatric Association, 2022; Schalock et al., 2010; Tassé, 2013).

Although adaptive behaviors and problem behaviors are two distinct and unrelated constructs that result in their own factor solution (Tassé, 2013)—i.e., the presence of poor acquisition of adaptive behaviors does not in itself lead to an increase in problem behaviors—the manifestation of maladaptive/problem behaviors seems to be prevalent among populations with intellectual disability compared to those with typical development, reaching as high as 63.9% among people with intellectual disability (Mastilo & Ćalasan, 2020). For example, maladaptive sexual behaviors can occur due to a poor understanding of the difference between public and private parts of the body or public and private places (Thompson & Brown, 1997). A person with intellectual disability may have difficulty grasping the difference between a school bathroom and a home bathroom because they are very similar in their bathroom fixtures, and the individual might not understand that masturbating in a school bathroom can be a crime, while masturbating in a home bathroom may be perfectly appropriate (Gadd, 2021).

Various theoretical orientations of treatment (e.g., psychoanalytic, biomedical, behavioral, etc.) and behavioral procedures (e.g., positive reinforcement or positive punishment or both, shaping procedure, respondent conditioning, etc.) have been used to address maladaptive sexual behaviors (Cicero, 2019), particularly the inappropriate or excessive masturbation that most frequently plagues family members, educators, and generally caregivers of people with intellectual disability (for a comprehensive review of the existing literature on this subject, see Cicero, 2019; Mann & Travers, 2020; Medina-Rico et al., 2018). Health interventions based on pharmacological treatment aim to reduce sexual maladaptive behavior by administering mirtazapine (Albertini et al., 2006) or amisulpride for the reduction of depressive symptoms (Chen et al., 2016), or leuprorelin for sexual libido reduction (Park et al., 2014). However, pharmacological treatments—in addition to raising ethical issues concerning informed consent and contravening the principles of the CRPD and WHO initiatives—tend to suppress libido and not change the maladaptive sexual behaviors that recur as soon as pharmacological therapy ceases (Walsh, 2000).

Unlike pharmacological treatments, behavioral (non-pharmacological) interventions aim not so much at eliminating inappropriate behaviors but reinforcing adaptive behaviors by teaching behavioral patterns (e.g., scripts; Schank & Abelson, 1977) to achieve effective and satisfying masturbation. For example, a strategy based on consequences (Lustig et al., 2014) differs in that it reinforces the frequency or duration of other appropriate behaviors rather than limiting inappropriate behaviors, which are difficult to suppress because they are reinforced by automatic processes that are difficult for educators to control. The strategy based on antecedents also facilitates the reinforcement of positive behavior. Nevertheless, it can be considered a preventative intervention because the change occurs before the problem occurs (Cihak et al., 2007). Indeed, according to the findings by Cihak et al. (2007), an antecedent-based intervention of self-directed auditory prompts for high school students with moderate to severe intellectual disability was as effective as or better than a response-based intervention. Positive results were also found with intervention models that combined one or more antecedent, instructional, and/or consequence strategies (Fisher et al., 2000; Wong et al., 2015).

The purpose of this article is to provide a case report on the efficacy of an expressive existential psychoeducational intervention (EEPI; Federici et al., 2019, 2020, 2021) to contain maladaptive sexual behaviors, and to highlight how EEPIs differ from other types of interventions, such as pharmacological treatment-based and cognitive–behavioral-based interventions. EEPIs combine informational (educational) content with exercises and psychotherapy techniques. Content can be delivered through videos and pictures based on the premise that observational learning serves both an informational and a motivational function (Bandura, 1986). In this case report, the implemented psychotherapy techniques were borrowed from Gestalt therapy (Perls et al., 1951), emotion-focused therapy (Elliott & Greenberg, 2017), and cognitive–behavioral therapy (Hollon & Beck, 2013). EEPIs for people with intellectual disability aim at the containment of sexual desire, not in the sense of suppression, but by actively managing the expression of one’s sex drive and affectivity that promotes the acquisition of adaptive behaviors in order to reduce the risk of problematic sexual behavior (e.g., exclusion from school and social life). EEPIs are conducted by psychologists who are licensed to practice and have received specific training at the undergraduate and postgraduate levels in disability and sexuality, as well as in neuropsychological assessment of neurodevelopmental disorders. Moreover, psychologists must have acquired expertise in the psychotherapeutic techniques of Gestalt therapy and cognitive–behavioral therapy. EEPIs differ from interventions based on the cognitive–behavioral model and a pharmacological treatment-based approach in that they integrate the former with Gestalt psychotherapy and emotion-focused therapy. In contrast to the pharmacological treatment-based approach, EEPIs prioritize the expression of emotions over containment, while also avoiding the use of pharmacological libido suppression.

In this report, an EEPI was used to reduce the maladaptive sexual behavior of a 17-year-old high school student with Trisomy 21 syndrome. The student exhibited inappropriate sexual behavior at school and in public, including oppositional and defiant behavior toward teachers and peers, the use of feces to insult and threaten, and disrespect for interpersonal boundaries with female classmates.

## Method

Our study’s method was based on a prospective longitudinal assessment of a single case, using a descriptive approach in the evaluation of the effectiveness of an intervention through the analysis of qualitative data in the form of a structured interview and the comparison of certain scores obtained by outcome measures at times t0, t1, and t2. The case study is a narrative report according to the Case Report guidelines (Gagnier et al., 2013).

### Case Presentation

A written informed consent to treatment and to the publication of the study were provided by parents of the patient (G) in a face-to-face consensus meeting. G was informed of the purpose of the treatment and encouraged to participate after obtaining parental consent. G clearly expressed his willingness to participate verbally. G was referred to LoveLife APS by his neuropsychiatrist of the public local health agencies of Perugia (National Health Service, Region Umbria, Italy) because G’s behavior was particularly difficult to manage, requiring specialized sexuality and disability professionals not present in the local health agency. LoveLife APS (www.love-life.it) is an Italian association of psychologists for the promotion of sexual health. The association offers to parents, educators, caregivers, and boys and girls with or without intellectual or motor disabilities practical help in managing masturbation and the menstrual cycle, preparing for menarche and puberty, and experiencing emotional and sexual relationships.

LoveLife APS does not offer sexual assistance services, as this is not permitted by Italian law, nor by the code of ethics of the Order of Psychologists to which the professionals working at LoveLife APS are registered, as are the authors themselves. However, LoveLife APS recognizes the importance of the issue of sexual assistance as a possible guarantee of access to sexual rights for people living with disabilities (Benoit et al., 2023a) and is committed to further research on this understudied topic (Benoit et al., 2023b).

Following his referral, G’s parents contacted LoveLife APS by scheduling a first interview that was conducted by SF. The first interview focused on collecting G’s medical history and neuropsychiatric diagnosis data and his parents’ requests.

G is a 17-year-old boy affected by Trisomy 21 syndrome (ICD-10, WHO, 2004: Q90) with moderate intellectual disability and IQ of 42. His medical diagnosis did not report cryptorchidism (ICD-10: Q53.9), hypoplasia of testis and scrotum (ICD-10: Q55.1), or hypoplasia of penis (ICD-10: Q55.6). During the anamnesis interview, the parents stated that G did not reach orgasm through masturbation and did not exhibit seminal fluid leakage during nocturnal emissions, although he manifested regular arousal and turgescence of the penis. In addition, the parents reported that they did not know the exact frequency or duration of G’s masturbatory activity. Parents reported G’s exhibitionistic behavior in school and social contexts (e.g., classroom and scout meetings). After being reprimanded by teachers, adults, and peers, G expressed oppositional feelings of anger in response to the frustration of the prohibition.

His intellectual development and adaptive behavior disorder affected his conceptual, social, and practical skills. The LoveLife APS psychologists subsequently constructed a functional profile of G based on ICF classification codes to highlight the patient’s strengths and weaknesses. All codes are provided in Supplementary Material, Table S1.

G has a moderate impairment of intellectual functions (b117.2) as a consequence of Trisomy 21 (ICD-10: Q90), with impairment of cognitive functions of attention (b140.3), memory (b144.2), and language (b167.3), and impairment of emotional (b152.3) and social (b122.2) functions. He also presented with impaired fine motor (b147.2) and sexual (b640.3) functions. In addition, G appeared to require restrictions on participation in social activities (d910.44) and demonstrated low performance in interpersonal interactions (d720.43). In addition, his masturbation was impaired due to his inability to use fine motor coordination of his fingers because of unusual upper extremity structure (s7309.4). Despite these critical issues, G has demonstrated an excellent capacity for daily autonomy (d230.02), cleanliness (d5100.00), and care (d520.00) of his body and a good level of reading and writing (d140.01) based on his record of scholastic performance (d820.23). From the analysis of the qualifiers related to the activity and participation domain codes, it appeared that G had developed good compensatory strategies (performance) with respect to his capacities in learning and applying knowledge (d1) and self-care (d5). This suggested to the psychologists that G, in a supportive environment and with appropriate facilitators, could improve his adaptive functioning. Conversely, G showed more difficulties in interpersonal interactions and relationships (d7) and community, social, and civic life (d9) compared to his potential. This restriction in social participation could be explained by poor family support (c310 + 1) and high friend barriers (c320.4).

Already in the first interview, the request of G’s parents was collected, which focused on G’s acquisition of skills related to adaptive sexual behavior concerning: (1) an appropriate management of masturbation; (2) distinguishing a public place from a private one; (3) the management of aggression and oppositional behavior, which manifested as destruction of personal and others’ belongings, in order to avoid exclusion from school; and (4) increasing his quality of sexual, emotional, and social life. Our subsequent intervention was based on the EEPI approach (Federici et al., 2019, 2020), which conveys educational content regarding skills to be acquired related to dimensions of adaptive behavior and behaviors to be avoided because they are socially unacceptable or unhealthy, therewith reinforcing the patient’s psychological maturation. Our intervention was designed to promote creativity and the expression of emotions by resorting to the use of Gestalt psychotherapy techniques in the existential context of the here and now of the helping relationship between patient and professional in order to increase awareness and responsibility.

The employed EEPI cycle was structured in nine steps: (1) contact and request; (2) anamnestic clinical interview; (3) patient’s first acquaintance meeting; (4) discussion of the request and designation of professionals; (5) projecting the intervention plan; (6) proposal of the intervention plan to parents or caregivers; (7) intervention; (8) return of the intervention to parents or caregivers; and (9) follow-up evaluation (about 3 months after intervention).

Steps 1 and 2 were accomplished in the first interview with G’s parents, which was followed by the first meeting with G (Step 3) on the basis of which the functional profile of G was drawn up by interviewer (SF). This was used by LoveLife APS staff to discuss and process the parents’ request and designate two psychologists, one male (EM) and one female (MER), who drew up the intervention plan and took charge of G, according to the EEPI cycle (Steps 6 to 9). During the initial meetings, G demonstrated a sexual interest in the female psychologist, which manifested as sexual requests and approaches. These were effectively contained by the therapist herself. The psychologists demonstrated competence in containing G’s sexual behavior without blaming him. They then worked on fostering awareness of the boundaries between the client’s (G) desire and the role of the professional. The intervention plan used two tools to both assess G’s sexual knowledge and behavior and indicate the intervention’s effectiveness, including: (1) the Italian adaptation of the Vineland-II Survey Interview Form (Balboni et al., 2016; Sparrow et al., 2005) and (2) Fraser and Dixon’s (2010) Sexual Knowledge and Behavior Assessment Tool (SKBAT).

The Vineland-II Survey Interview allows the assessment of adaptive behavior of individuals aged between 0 and 90 years. The measurement consists of four domains and 11 subdomains, and an additional index that pertains to maladaptive and critical behaviors.

The four domains and relative subdomains (within brackets) are the following: Communication (Receptive, Expressive, and Written), Daily Living Skills (Personal, Domestic, and Community), Socialization (Interpersonal Relationships, Play and Leisure Time, and Coping Skills), and Motor Skills (Gross and Fine). In the first administration of the Vineland-II, all scales and their subscales were used for assessment purposes to gain a clear idea of G’s strengths and weaknesses with respect to the efficacy of the EEPI treatment.

On the other hand, in the post-intervention administration (t1) and follow-up (t2; about 3 months after intervention) stages, the only scale that was used to assess the effectiveness of the EEPI was the Socialization scale and its subscales Interpersonal Relationships, Play and Leisure Time, and Social Rules. The sum of the scores obtained from each subdomain was compared with the equivalent age values provided by the normative scores of the Italian adaptation (Balboni et al., 2016).

The Maladaptive Behavior indexes consist of: (1) the Maladaptive Behavior Index (36 items), composited of Internalizing, Externalizing, and other types of undesirable behaviors that may interfere with the individual’s adaptive functioning; and (2) the Maladaptive Behavior Critical Index (14 items), pertaining to more severe maladaptive behaviors that can provide clinically important information. The Maladaptive Behavior domain was used to assess the presence/absence of problematic behaviors based on frequency, where 0 = never performed, 1 = performed sometimes, and 2 = usually or habitually occurs. The Maladaptive Behavior indexes do not possess Italian normative reference scores. The indexes were used for measuring changes in G’s obvious undesirable behaviors by comparing the pre-treatment (t0) scores from each subdomain at the end of treatment (t1) and after the follow-up assessment (t2).

The Vineland-II interview was conducted by psychologists EM and MER during three interviews with G’s parents in the following order: t0, after the presentation of the intervention project to the parents (Step 6); t1, at the end of treatment (Step 7); and t2, at the follow-up assessment (Step 9). The subdomains of Communication, Daily Living Skills, and Motor Skills were only administered at t0 under the assumption that they were not the target of the intervention and would not be affected by the intervention.

Fraser and Dixon’s (2010) SKBAT was adapted into Italian by Laoreti and Federici (2023) following Beaton et al.’s (2000) guidelines for the process of cross-cultural adaptation of self-report measures. The tool, as a structured interview, assesses the level of sexual knowledge and understanding of people with intellectual disability before (t0) and after (t1) a sexual and relationship education program and on body parts, masturbation and sexual intercourse, pregnancy and parenting, and sexually transmitted infections, including condom use. The assessment tool includes a checklist for parents to complete and a questionnaire, accompanied by illustrations. Each of the four sections (about the patient and others, sexual behavior, looking after others, sexual health) is introduced by a picture, arranged in ascending levels of symbolic difficulty. Each picture is accompanied by a question, below which is the respondent’s potential answer (G). Psychologists EM and MER administered the SKBAT to G during initial (t0) and final intervention sessions (t1), at Step 7.

### Therapeutic Intervention

The intervention (t0) was structured over 15 weekly sessions, each lasting approximately one hour. The sessions were divided into two phases, which were conducted at the LoveLife APS headquarters (Phase 1, Sessions 1 and 2; Phase 2, Sessions 6, 7, and 9), G’s home (Phase 1, Sessions 3–5; Phase 2, Sessions 1–5), and a youth recreation center (Phase 2, Sessions 8 and 9). Two weeks (t1) and three months (t2) following the conclusion of the intervention, the psychologists EM and MER conducted the Vineland-II interview with G’s parents. This was done to ascertain the long-term continuation of the goals achieved by G during the intervention phases. After the intervention, the two psychologists did not have any further meetings with G.

The objective of each phase was as follows:

- Phase 1, five sessions: to improve masturbation management to reduce inappropriate sexual behaviors; and
- Phase 2, 10 sessions: to acquire more social–affective skills to appropriately identify, express, and manage anger.

### Phase 1: Improving Masturbation Management and Reducing Inappropriate Sexual Behaviors

The (psycho)educational content in this phase included the identification of problem behaviors (according to the Maladaptive Behavior Index) and their management. The psychotherapeutic techniques used were based on a behaviorist approach according to the paradigms of classical and operant conditioning, in which the sexual aids used (masturbation “kits”) acted as a conditioned stimulus to induce the sequence of correct behaviors. Repetition of these behaviors and reinforcement through the achievement of orgasm allowed the learned adaptive behavior to be consolidated and the maladaptive behaviors to be extinguished. The underlying hypothesis was that inappropriate sexual behaviors would be reduced if G learned to functionally and satisfactorily manage his masturbation, thereby channeling the adolescent sex drive into autoeroticism.

The first session was aimed at learning the concepts of public and private body parts and the ritualization of masturbation behavior in the patient’s room. Anatomical models of latex genitalia were used, and the patient was asked to verbally describe them anatomically and functionally. For example, G was asked to describe the difference between seminal fluid and urine. A masturbation kit was used for demonstration purposes: a beauty case containing some products that can be used for proper masturbation and personal hygiene, including wipes, mild soap, lubricating gel, towel, disinfectant gel, and a “do not disturb” sign to be hung on the primary door handle before the practice begins.

In the following four sessions, in order to consolidate the learning “scripts” (Schank & Abelson, 1977), namely the predetermined sequences of actions that define functional and satisfactory masturbation in a private room, the sequence of procedures before and after masturbation was always repeated: (1) hang the “do not disturb” sign on the outside door handle; (2) close the door; (3) undress; (4) fold the underwear and place it on the chair next to the bed; (5) apply lubricant to the penis; (6) practice masturbation; (7) wipe off with wipes; (8) put on clothes; (9) remove the “do not disturb” sign from the door; (10) go to the bathroom; (11) wash the penis and hands with mild soap and water; (12) dry with the special towel; and (13) disinfect hands with disinfectant gel. Since G was unable to perform proper hand movements on his penis due to impaired upper motor skills, and thus could not achieve orgasm and ejaculation, a sexual aid, a “blow cup,” was introduced as early as the third session. In addition, multisensory experiences (background music, blindfold, use of fetish clothing chosen by G) were associated with increase pleasure and arousal.

### Phase 2: Acquiring Social–Affective Skills to Identify, Express, and Manage Anger

In the second phase and its 10 sessions, the intervention focused on the implementation of social–affective skills, including anger recognition and the management of aggressive and destructive behaviors. In this phase, the EEPI included the use of Gestalt psychotherapy techniques and expressive tools, including emoticons, artistic mediators, pictographic symbols, and graphic vignettes, some created using Boardmaker® software (by Mayer-Johnson) and others adapted from Picture Yourself 1 and 2 (Dixon, 2006, 2014) and Sex and the 3Rs (McCarthy & Thompson, 2016).

In the first five sessions, G was guided to understand the social consequences of maladaptive and socially unacceptable behaviors and to recognize his own sadness and loneliness as a result of social isolation. These same goals were then reinforced through the use of role-playing techniques, modeling, and a transactional object. This was a red pillow called “the anger pillow,” used to reinforce adaptive and cathartic responses to anger expression. Role-playing techniques and live demonstrations were employed through a modeling approach, wherein therapists exhibited the appropriate sequences of behavior and had G replicate them in a repetitive manner. Regarding the acquisition of learning sequences, G demonstrated no difficulties. Regarding the awareness of the functionality of these acquisitions in everyday life, G demonstrated difficulties in learning. Consequently, symbolic techniques and tools (AAC and artistic mediators) were employed to facilitate consolidation. In the sixth and seventh sessions, the intervention focused on communicative proxemics, body boundaries, and a person’s consent. In the eighth and ninth sessions, G was accompanied during playful recreational activities in a peer group of adolescents with intellectual disability, where he was challenged to communicate and respect boundaries, with the goal of transferring established learning to real-life situations. In the final session (10), the work focused on the themes of a person’s consent and physical contact (hug), using a second transitional object, a white pillow called “the hug pillow.” The session ended with the use of the expressive technique of collage, with the creation of a poster board divided into two parts, one representing G child and the other G adult, with the aim of accompanying him toward an integration to awareness of his psychosexual development path.

## Results

All underlying data supporting the results of the study are included in Supplementary Material.

### Vineland-II

As shown in Table 1, G’s adaptive behavior was low in all domains and subdomains, except for the Domestic subdomain, which was moderately low. The Personal subdomain score and the Fine Motor subdomain score came out significantly low. After the EEPI (t1), the v-scale and age-equivalent scores improved in all subdomains, although not enough to improve the adaptive level.

**Table 1:** Vineland-II adaptive behavior domains and subdomains scores by three interviews (t0, t1, and t2). The adaptive level (AdL) describes the level of G’s adaptive behavior through five ordered categories: low, moderately (mod.) low, adequate, mod. high, and high

	t0			t1			t2		
Domain	RS (V-S)	AdL	AgE	RS (V-S)	AdL	AgE	RS (V-S)	AdL	AgE
Communication									
Receptive	27(8)	Low	3:8						
Expressive	63(6)	Low	3:1						
Written	26(5)	Low	6:10						
Daily living skills									
Personal	45(2)	Low	3:9						
Domestic	23(11)	Mod. Low	10:3						
Community	47(8)	Low	7:10						
Socialization									
Interp. relation	31(2)	Low	2:6	39(3)	Low	3:5	39(3)	Low	3:5
Play and leisure time	28(3)	Low	3:6	30(5)	Low	3:8	30(5)	Low	3:8
Coping skills	17(2)	Low	5:6	31(4)	Low	6:5	31	Low	6:5
Motor skills									
Gross	41		1:8						
Fine	18		0:11						

RS, raw score; V-S, v-scale score; AdL, adaptive level; AgE, age equivalent

As shown in Table 2, in all domains and subdomains there was an apparent decrease in scores after the EEPI (t1), which also appeared stable at follow-up (t2).

**Table 2:** Vineland-II maladaptive behavior indexes and subdomains scores by three interviews (t0, t1, and t2)

	t0	t1	t2
Domain	raw score	raw score	raw score
Maladaptive Behavior Index	37(72)	20(72)	20(72)
Internalizing	6(22)	4(22)	4(22)
Externalizing	14(20)	5(20)	5(20)
Other undesirable behavior	17(20)	11(30)	11(30)
Maladaptive Behavior Critical Index	14(14)	10(14)	10(14)

Table 2 shows the raw scores obtained from the semi-structured Vineland-II interview administered to G’s parents. The scores obtained were summed to obtain raw scores. In the same column where the raw scores are inserted, the v-scale score is reported in parentheses (i.e., the weighted score with normal distribution, with mean = 15 and SD = 3, describes the individual’s performance in units of SDs compared to the normative age group). The equivalent age, reported below, is the adaptive behavior score in units of years and months compared to the mean development of the same behavior in the normative group representative of the general reference population. All underlying data supporting the results of the study are included in Supplementary Material (Table S2 and S3) to the main text.

### Sexual Knowledge and Behavior Assessment Tool

According to the SKBAT interview conducted at t0, G showed that he knew a general difference between girls’ and boys’ bodies, distinguishing male and female genital organs. He also named the other parts of the human body appropriately (hair, hands, arms, etc.). However, G had difficulty identifying where feces come from and naming several parts of the female body. This seemed to be due more to initial embarrassment, due to an internalized feeling of shame, than actual ignorance, since at t1 G demonstrated accurate knowledge about them.

Regarding nocturnal emissions depicted in a picture (Supplementary Material, Table S4, picture 10, questions 54 to 57), to the question “Tell me what is happening in this picture” (picture 10), at t0 G replied, “pee and liquid,” while at t1 he recognized and named the seminal fluid calling it “sperm.” To question 57, “At about what age do most boys start to have wet dreams?,” G reply confused dreams with fantasy: at t0 “ham and socks” and at t1 “I undress naked in class and smear poop on desks,” which turned out a stereotypical exclamation and inappropriate behavior associated with thoughts about something sexual.

With respect to penile erection and its function (Supplementary Material, Table S4, picture 11, questions 58 and 59), G showed enriched knowledge after the intervention. In fact, at t0, G responded to question 58, “What is it called when a boy’s or man’s penis looks like this [penile erection]?” with the slang term “dick.” At t1, G was able to articulate a much richer response: “[When] the penis gets hard, I put on my socks and touch my feet so that the fluid comes out of the penis,” demonstrating that he was linking erotic fantasy to unproblematic behavior.

Also regarding the act of masturbation, G showed that he had acquired a richer representation after the intervention. In response to question 84, “Tell me about this picture,” associated with the picture of a man masturbating (Supplementary Material, Table S4, picture 16), G replied at t0, “He is rubbing his dick,” which turns out to be the only way he touches his genitals. Then, at t1, he responded, “He does the exercises,” referring to sequences of actions (“exercise”) that precede and follow masturbation, as learned during the intervention. To question 86, “What happens when he masturbates/wanks/does private touching?,” also associated with picture 16, G did not answer at t0, showing a lack of understanding of the term “private.” Then, at t1, G responded, “I do the exercises so I can see the stars!,” demonstrating not only an understanding of the association between masturbation and a private place, but also experienced functional masturbation and achieved orgasm. The same cognitive and behavioral maturation seems to be clearly achieved by his answers to question 88, “Is it OK to do this in a private place?,” which G had not answered at the first interview (t0), while at the end of the intervention (t1) he answered, “I can only do the exercises in the bedroom.”

The findings from the qualitative interview data on sexual knowledge and behaviors clearly show G’s progress and achievement due to the EEPI treatment. G both understood that masturbation should only occur in private spaces and learned how to achieve orgasm and ejaculation. To see all of G’s responses to SKBAT before and after the intervention, see Supplementary Material, Table S4.

## Discussion

The present case study showed how an EEPI (expressive existential psychoeducational intervention) (Federici et al., 2019, 2020, 2021) was applied in order to reduce the maladaptive sexual behaviors of a 17-year-old high school student, G, with Trisomy 21 syndrome, who exhibited oppositional and challenging behaviors toward teachers and peers, thus exposing him to the risk of exclusion from school and social life. In a prospective longitudinal assessment on a single case, through the analysis of qualitative data, the present case study focused on highlighting the effectiveness of an EEPI to contain G’s maladaptive sexual behaviors based on the changes that emerged in specific adaptive and maladaptive domains, as assessed by the Vineland-II and SKBAT interviews administered before and after the intervention. Specifically, our EEPI was designed to improve G’s behavior in three problematic areas of his sexual behavior (inappropriate masturbation, not distinguishing between public and private places, and aggression and oppositional behavior) in order to increase the quality of his sexual, emotional, and social life.

Results showed the effectiveness of the EEPI in reducing all three of G’s problem behaviors. As indicated by the Maladaptive Behavior Index, G improved his behavior related to overconfidential attitudes toward others. At the same time, in terms of anger and aggression management, we observed that G was controlling his anger and not destroying his own and others’ belongings. Finally, in relation to the management of effective and functional autonomous masturbation, G acquired a good capacity for appropriate sexual behaviors (item 22) by understanding the association between masturbation and a private place, experiencing functional masturbation and achieving orgasm (G’s “I do the exercises so I can see the stars”). Therefore, based on the scores that emerged, it is possible to say that the EEPI showed good effectiveness in limiting G’s maladaptive sexual behaviors.

Furthermore, the fact that there was no substantial change observed in terms of adaptive behaviors (Table 2), but there was a reduction in maladaptive behaviors, confirms the independence of the adaptive behavior construct from the maladaptive behavior construct (Tassé, 2013). Although there was no direct correlation between the improvement in adaptive behaviors and the reduction in maladaptive behaviors, it is clear that the reduction in maladaptive behaviors was protective for G, providing cognitive and emotional resources to improve his quality of life by neutralizing barriers to sociality and autonomy.

Regarding the benefits of applying the EEPI model (Federici et al., 2019, 2020, 2021) with G, some strengths emerged when compared with the cognitive–behavioral (Cihak et al., 2007; Fisher et al., 2000; Lustig et al., 2014; Schank & Abelson, 1977; Wong et al., 2015) and pharmacological treatment-based models (Albertini et al., 2006; Chen et al., 2016; Park et al., 2014). With respect to the interventions based on the cognitive–behavioral model, the EEPI makes many of its techniques its own, enriching them with expressive techniques from Gestalt psychotherapy (Perls et al., 1951) and techniques from emotion-focused therapy (Elliott & Greenberg, 2017). This integration reinforces a psychoeducational approach that focuses less on containment and more on the expression of emotions.

In contrast to the pharmacological treatment-based approach, instead, EEPIs are equally effective in reducing problematic sexual behavior without suppressing libido, but rather redirecting it toward full and healthy satisfaction through the acquisition of socially acceptable sexual acts that improve the individual’s overall quality of life, with full ethical respect for the individual. In addition, the consolidation of adaptive and satisfying sexual behaviors shows longer-term effectiveness beyond treatment, as evidenced by G’s behaviors observed at follow-up, unlike drug treatments whose effectiveness ends when therapy is discontinued (Walsh, 2000).

However, EEPIs have cost and time disadvantages compared to psychiatric drug treatments. In fact, while the cost of a psychiatric–pharmacological treatment would be covered by the Italian National Health System, the psychological treatment offered with an EEPI would be minimal and, given the duration of treatment (3 to 6 months), much more expensive than a psychiatric drug. In addition, long-term treatments, such as EEPIs, may conflict with a school’s urgency, as in G’s case, for example, to resolve serious school integration problems resulting from a student’s maladaptive behavior. The risks of school exclusion and social marginalization may lead parents of a student with intellectual disability to prefer short-term interventions, including pharmacological treatments, rather than face the long timeline of psychological and educational treatments involving both the individual and the family system.

## Data Availability

Data on Vineland-II interviews (t0, t1, t2) and SKBAT responses before and after the intervention are available in Supplementary Material.
